# The Effect of Lyso-PAF on Ciliary Activity of Human Paranasal Sinus Mucosa *in vitro*

**DOI:** 10.1155/S0962935194000074

**Published:** 1994

**Authors:** T. Ganbo, K. Hisamatsu, H. Inoue, T. Nakajima, Y. Murakami

**Affiliations:** 1 Department of Otolaryngology Yamanshi Medical University 1110 Shimogato Tamaho-cho, Nakakoma-gun Yamanashi 409-38 Japan; 2 Department of Plastic and Reconstructive Surgery St Marianna University School of Medicine Kawasaki Kanagawa Japan

## Abstract

The effect of lyso-PAF on ciliated cells was investigated *in vitro*. Normal mucosa was surgically obtained from human paranasal sinuses and incubated in the form of tissue culture. Ciliated epithelium was magnified under an inverted microscope, and ciliary movement was photo-electrically measured. Ciliary activity was significantly inhibited by 10^−8^ M lyso-PAF and could be restored. The effect of lyso-PAF was completely blocked by CV-6209, a specific PAF antagonist. The PAF concentration in the incubation medium of lyso-PAF was determined by radioimmunoassay, because PAF is a well known inhibitor of ciliary activity. PAF gradually increased and after 20 min reached its maximal level. These findings indicated the existence of an enzyme in the paranasal sinus mucosa, by which lyso-PAF is converted to PAF, and that lyso-PAF can inhibit ciliary activity by means of PAF.


Mediators of Inflammation 3, 41–44 (1994)

### The effect of lyso-PAF on ciliary activity of human paranasal sinus mucosa *in vitro*

T. Ganbo,[1,CA] K. Hisamatsu,[1] H. Inoue,[2] T. Nakajima[1] and Y. Murakami[1]

[1]Department of Otolaryngology, Yamanshi Medical University, 1110 Shimogato, Tamaho-cho, Nakakoma-gun, Yamanashi 409-38, Japan; [2]Department of Plastic and Reconstructive Surgery, St Marianna University School of Medicine, Kawasaki, Kanagawa, Japan

[CA] Corresponding Author

**THE effect of lyso-PAF on ciliated cells was investigated *in vitro*. Normal mucosa was surgically obtained from human paranasal sinuses and incubated in the form of tissue culture. Ciliated epithelium was magnified under an inverted microscope, and ciliary movement was photo-electrically measured. Ciliary activity was significantly inhibited by $10^{-8}$ M lyso-PAF and could be restored. The effect of lyso-PAF was completely blocked by CV-6209, a specific PAF antagonist. The PAF concentration in the incubation medium of lyso-PAF was determined by radioimmunoassay, because PAF is a well known inhibitor of ciliary activity. PAF gradually increased and after 20 min reached its maximal level. These findings indicated the existence of an enzyme in the paranasal sinus mucosa, by which lyso-PAF is converted to PAF, and that lyso-PAF can inhibit ciliary activity by means of PAF.**

**Key words:** Acetyltransferase, Ciliary movement, Ciliated epithelium, Lyso-PAF, PAF, Radioimmunoassay

### Introduction

Platelet activating factor (PAF) is considered to be a potent chemical mediator which can induce various features in allergic and inflammatory disorders of the respiratory tract. Inhaled PAF causes rhinitis-like symptoms,[1] bronchial constriction and prolonged airway hyperresponsiveness.[2,3] PAF also induces release of mucus from tracheal submucosal glands[4] and dysfunction of ciliated cells[5] *in vitro*. However, lyso-PAF, which is a precursor and metabolite of PAF, is thought to have minimal effect compared with PAF. There are few reports detailing the effect of lyso-PAF on ciliary activity,. because it was considered to be inactive in the biological system. The authors observed ciliary activity of human paranasal sinus mucosa, which is a part of the respiratory epithelium, by incubating the mucosa with lyso-PAF. In this paper, the effect of lyso-PAF on ciliary activity is described and the cause of its effect is discussed.

### Materials and Methods

*Maintenance of human paranasal sinus mucosa:* Normal paranasal sinus mucosa was carefully removed by surgical procedure from the ethomidal sinuses of patients who suffered from facial trauma. The mucosa was rinsed in Eagle's minimal essential medium (MEM) to remove blood cells and mucus, and cut into pieces of approximately 4 mm × 4 mm with scissors. The mucosal specimens were examined by light microscopy, transferred onto a collagen layer in 35 mm × 10 mm culture dishes containing 1 ml of Eagle's MEM with 10% foetal calf serum (FCS), and incubated at 37°C under 5% $CO_2$ in a 100% humidified incubator. The culture medium was changed 24 h later to remove mucus and cellular debris, and every 48 h thereafter. These experiments were conducted with the understanding and consent of the patients and/or their families.

*PAF, lyso-PAF and PAF antagonist:* 1-*O*-hexadecyl-2-acetyl-*sn*-glycero-3-phosphorylcholine (PAF) and 1-*O*-hexadecyl-*sn*-glycero-3-phosphorylcholine (lyso-PAF) were used (Bachem Feinchemikalien AG, Bubendorf, Switzerland). PAF and lyso-PAF were dissolved in methanol at a concentration of $10^{-2}$ M and then diluted with Eagle's MEM to $10^{-8}$ M. CV-6209 (2-[*N*-acetyl-*N*-(2-methoxy-3-octadecyl-carbamoyl-oxy-propoxy-carbonyl) aminomethyl]-1-ethylpyridinium chloride (Takeda Chemical Industries Ltd, Osaka, Japan)), a highly specific PAF inhibitor,[6] was used. CV-6209 was dissolved in physiological saline at $10^{-3}$ M and then diluted with the medium. Each test solution consisted of 1.5 ml to which the mucosal specimens were exposed.

*Observation and recording of ciliary activity:* The mucosal specimen was irrigated three times with Eagle's MEM without FCS before exposure to each test solution. Ciliated cells on the mucosal epithelium, at 37°C under 5% $CO_2$, were magnified under an inverted microscope equipped with a camera, and observed on the TV monitor. Ciliary activity of each ciliated cell was recorded on video tape and photo-electrically measured by placing a 3 mm wide

© 1994 Rapid Communications of Oxford Ltd photo-sensor on each beating bundle of cilia which was displayed on the TV monitor.

*Radioimmunoassay of PAF:* The concentration of PAF in the culture medium was measured using a PAF[$^{125}$I] radioimmunoassay kit (E. I. DuPont de Nemours & Co., Boston, USA). All procedures were carried out using polypropylene pipette tips and polystyrene tubes for assay. To each tube, 100 μl of each sample to be tested and 100 μl of PAF primary antibody were added followed by a 15 min incubation period at room temperature, and then 100 μl of the secondary antibody/tracer solution were added followed by an 18 min incubation at room temperature. After the addition of 2 ml of assay buffer (50 mM sodium citrate buffer containing 0.1% sodium azide and 0.05% Tween 20, pH 6.3), the tubes were centrifuged at 2000 × *g* for 30 min at room temperature and then decanted. The remaining radioactivity was counted by the *r*-well counter. The concentrations of PAF in the test samples were determined from the calibration curve.

*Statistical analysis:* The significant difference between recorded values was statistically determined at $p < 0.01$ on the Student's *t*-test for unpaired data.

### Results

*Effect of lyso-PAF:* Five mucosal specimens obtained from five patients (A to E) were exposed to $10^{-8}$ M lyso-PAF. The time courses of the effect of $10^{-8}$ M lyso-PAF are illustrated in Fig. 1. Ciliary activity was inhibited after 1 h of exposure in Specimen A, 2 h in B, 5 h in C and D, and 7 h in E. There was a significant difference between the ciliary beat frequency during the initial activity (zero time) and its lowest level of activity in each specimen. However, the ciliary beat frequency in each specimen recovered from its lowest level of activity. Consequently, inhibition was temporary and reversible.

*Effect of CV-6209:* CV-6209, which is a specific PAF receptor antagonist, inhibited the effect of PAF and lyso-PAF on ciliary activity (Fig. 2). Significant ciliary inhibition was time dependently induced by $10^{-8}$ M PAF. When the mucosa was incubated with $10^{-8}$ M PAF and $10^{-6}$ M CV-6209 after a 15 min preincubation with $10^{-6}$ M CV-6209, ciliary activity showed no remarkable change for 10 h. However, a concentration of $10^{-8}$ M CV-6209 was not enough to inhibit the effect of $10^{-8}$ M PAF on ciliary activity. Ciliary activity was moderately inhibited by PAF when the mucosa was incubated with $10^{-8}$ M PAF and $10^{-8}$ M CV-6209 after a 15 min preincubation with $10^{-8}$ M CV-6209. When the mucosa was incubated with $10^{-8}$ M lyso-PAF, ciliary activity was significantly inhibited after 5 h of exposure and restored after 10 h. Furthermore, the effect of $10^{-8}$ M lyso-PAF on ciliary activity was completely blocked by $10^{-6}$ M CV-6209. In these experiments (Fig. 2), specimens from Patient C were used. The time course is illustrated in Fig. 1.

*Time course of PAF in the culture medium:* Assays of PAF in the incubation chamber, when the mucosa was incubated with $10^{-8}$ M PAF, were carried out. A time dependent decrease in PAF concentration in the tissue culture medium (Fig. 3) was observed. PAF concentration was halved within 11.4 min, and within 60 min, its concentration was only 3.2% of the initial concentration.

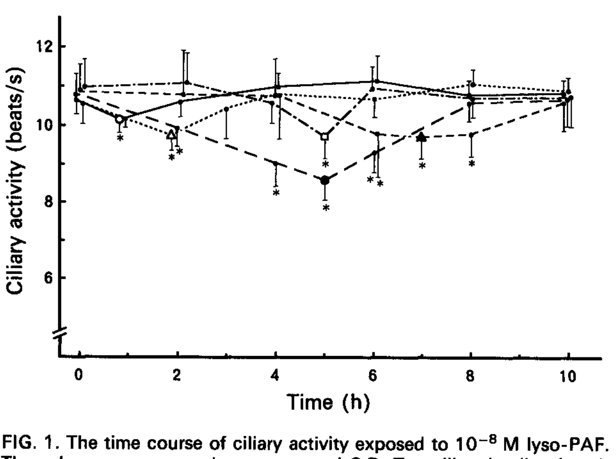

FIG. 1. The time course of ciliary activity exposed to $10^{-8}$ M lyso-PAF. The values are expressed as means and S.D. Ten ciliated cells of each specimen (A to E) were observed. *$p < 0.01$, compared with the initial ciliary activity., ○ Patient A; △, Patient B; □, Patient C; ●, Patient D; ▲, Patient E.

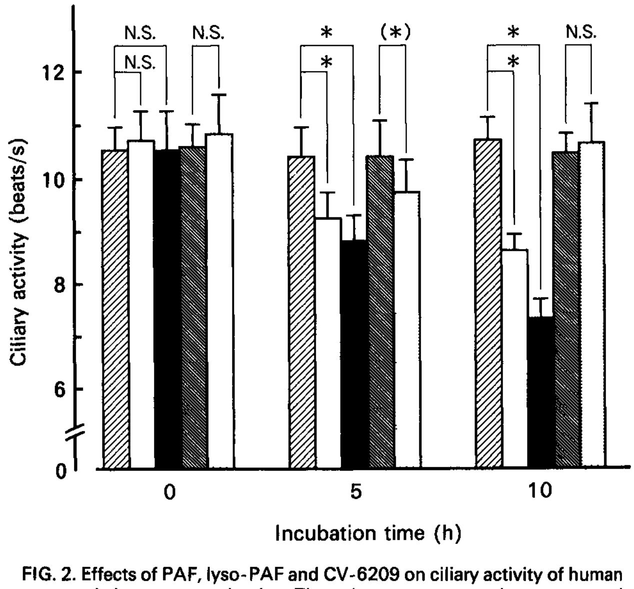

FIG. 2. Effects of PAF, lyso-PAF and CV-6209 on ciliary activity of human paranasal sinus mucosa *in vitro*. The values are expressed as means and S.D. $n = 10$. *, (*): $p < 0.01$. ▨, $10^{-6}$ CV-6209 + $10^{-8}$ M PAF; □, $10^{-8}$ M CV-6209 + $10^{-8}$ M PAF; ■, $10^{-8}$ M PAF alone; ▩, $10^{-6}$ M CV-6209 + $10^{-8}$ m lyso-PAF; ▤, $10^{-8}$ M lyso-PAF alone.

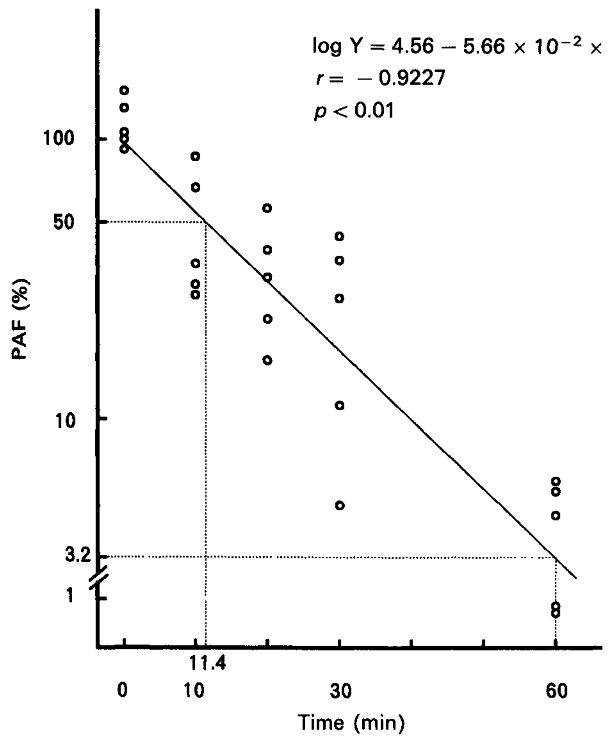

FIG. 3. The time course of PAF concentration in the incubation medium when the mucosa was incubated with $10^{-8}$ M PAF. $n = 5$.

*The concentration of PAF in the incubation medium of lyso-PAF:* Using radioimmunoassay, the concentration of PAF in the incubation medium was observed over time when the mucosa was incubated with $10^{-8}$ M lyso-PAF (Fig. 4). The concentration of PAF gradually increased and after 20 min, the mean of PAF concentration reached the maximum of $0.273 \times 10^{-8}$ M. However, PAF was not detected after 60 min.

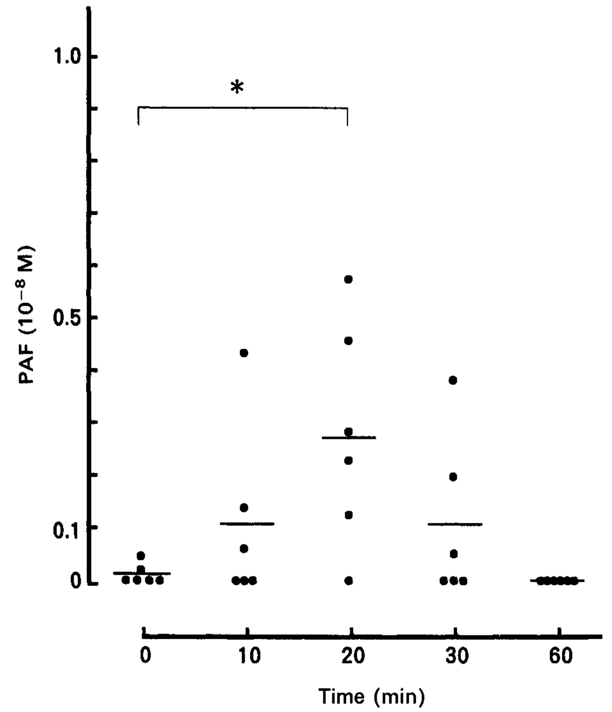

FIG. 4. The time course of PAF concentration in the incubation medium when the mucosa was incubated with $10^{-8}$ M lyso-PAF. The bars are expressed as means. $n = 6$. $^{*}p < 0.01$.

### Discussion

Ciliary activity was significantly inhibited by lyso-PAF and could be restored in all five specimens (Fig. 1). There were some differences in the time at which ciliary activity was inhibited in each specimen. The earliest was 1 h, the latest was 7 h, and the average of five experiments was 4 h. The inhibition in ciliary activity induced by lyso-PAF were irregular. However, the effect of lyso-PAF on ciliary activity was completely blocked by CV-6209, which is a specific PAF antagonist (Fig. 2). Furthermore, PAF was detected in the culture medium when the paranasal sinus mucosa was incubated with lyso-PAF (Fig. 4). The mean of PAF concentration reached its maximal level after 20 min incubation. There was a significant difference between the concentrations of PAF at the initial time (zero time) and at 20 min. These results indicate that lyso-PAF was converted to PAF, which then induced ciliary inhibition.

Acetyltransferase activity, which can change lyso-PAF to PAF, has been found in various rat tissues,[7] murine macrophages,[8] and human neutrophils[9] and eosinophils.[10] One of the enzymes in the paranasal sinus mucosa was supposed to produce PAF from lyso-PAF. The concentrations of secondary PAF in the culture medium were variable. The formation of PAF was suspected to depend on lyso-PAF as a substrate and the converting enzyme. The respective pieces of mucosal volume were not strictly constant and the activity of the converting enzyme in each mucosa might be variable. Therefore, the peak concentrations of newly generated PAF appeared to be variable. It is supposed that such various concentrations of secondary PAF induced the variety of the maximum of ciliary inhibition. The earlier the concentration at which PAF reached its peak, the earlier the inhibition in ciliary activity appeared. The higher the concentration of PAF, the stronger the inhibition of ciliary activity became. However, the concentration of PAF in the culture medium was rapidly reduced by half within 11.4 min, and within 60 min most of the PAF was diminished (Fig. 3). PAF could have an effect on ciliated cells for only a short period of time.

In a previous study[11] ciliary dysfunction is induced primarily within the first 60 min after exposure to PAF. PAF is unstable, and easily converted to an inactive metabolite. It is uncertain whether the reduction of PAF in the medium was achieved enzymatically by acetylhydrolase[12] or non-enzymatically by environmental agents, such as temperature and light. In this study, the PAF formed by conversion of lyso-PAF and detected in the culture medium decreased rapidly, and ciliary inhibition was reversible.

PAF is one of the most important mediators which can induce mucosal damage and respiratory hyperresponsiveness. It may be necessary to investigate the more detailed mechanisms of the conversions between lyso-PAF and PAF. However, the findings obtained from the present study support the proposal that an enzyme which converts lyso-PAF to PAF exists in human paranasal sinus mucosa. Furthermore, lyso-PAF is secondarily capable of inhibiting ciliary activity.

### References

1. Misawa M, Iwamura S. Platelet-activating factor (PAF)-induced rhinitis and involvement of PAF in allergic rhinitis in guinea pigs. *Japan J Pharmacol* 1990; **54**: 217–226.
2. Cuss FM, Dixon CMS, Barnes PJ. Effects of inhaled platelet activity factor on pulmonary function and bronchial responsiveness in man. *Lancet* 1986; **ii**: 189–192.
3. Chung KF, Aizawa H, Leikauf GD, Ueki IF, Evans TW, Nadel JA. Airway hyperresponsiveness induced by platelet-activating factor: role of thromboxane generation. *J Pharmacol Exp Ther* 1986; **236**: 580–584.
4. Hahn HL, Purnama I, Lang M, Sannwald U, Stenzel H. Effects of platelet activating factor on release of mucus from tracheal submucosal glands in the ferret. *Am Rev Respir Dis* 1985; **131**: A27.
5. Ganbo T, Hisamatsu K. Mucosal dysfunction and damage induced by platelet activating factor (PAF). *Acta Otolaryngol (Stockh)* 1990; **110**: 427–436.
6. Terashita Z, Imura Y, Takatani M, Tsushima S, Nishikawa K. CV-6209, a highly potent antagonist of platelet activating factor *in vitro* and *in vivo*. *J. Pharmacol Exp Ther* 1987; **242**: 263–268.
7. Wykle RL, Malone B, Snyder F. Enzymatic synthesis of 1-alkyl-2-acetyl-*sn*-glycero-3-phosphocholine, a hypotensive and platelet-aggregating factor. *J Biol Chem* 1980; **255**: 10256–10260.
8. Ninio E, Mencia-Huerta JM, Heymans F, Benvenisite J. Biosynthesis of platelet-activating factor. I. Evidence for an acetyltransferase activity in murine macrophages. *Biochim Biophys Acta* 1982; **710**: 23–31.
9. Alonso F, Gil MG, Sanchez-Crespo M, Mato JM. Activation of 1-alkyl-2-lyso-glycero-3-phosphocholine:acetyl-CoA transferase during phagocytosis in human polymorphonuclear leukocytes. *J Biol Chem* 1982; **257**: 3376–3378.
10. Lee T, Malone B, Wassermann SI, Fitzgerald V, Snyder F. Activities of enzymes that metabolized platelet-activating factor (1-alkyl-2-acetyl-*sn*-glycero-3-phosphocholine) in neutrophils and eosinophils from humans and the effect of a calcium ionophore. *Biochem Biophys Res Commun* 1982; **105**: 1303–1308.
11. Ganbo T, Hisamatsu K, Nakazawa T, Kamigo A, Murakami Y. Platelet activating factor (PAF) effects on ciliary activity of human paranasal sinus mucosa *in vitro*. *Rhinology* 1991; **29**: 231–237.
12. Blank ML, Lee T, Fitzgerald V, Snyder F. A specific acetylhydrolase for 1-alkyl-2-acetyl-*sn*-glycero-3-phosphocholine (a hypotensive and platelet-activating lipids). *J Biol Chem* 1981; **256**: 175–178.

**Received 26 August 1993;**
**accepted in revised form 18 November 1993**

## References

[PDF_00342] Alonso F., Gil M. G., Sánchez-Crespo M., Mato J. M. (1982). Activation of 1-alkyl-2-lysoglycero-3-phosphocholine. Acetyl-CoA transferase during phagocytosis in human polymorphonuclear leukocytes.. J Biol Chem.

[PDF_00354] Blank M. L., Lee T., Fitzgerald V., Snyder F. (1981). A specific acetylhydrolase for 1-alkyl-2-acetyl-sn-glycero-3-phosphocholine (a hypotensive and platelet-activating lipid).. J Biol Chem.

[PDF_00325] Chung K. F., Aizawa H., Leikauf G. D., Ueki I. F., Evans T. W., Nadel J. A. (1986). Airway hyperresponsiveness induced by platelet-activating factor: role of thromboxane generation.. J Pharmacol Exp Ther.

[PDF_00322] Cuss F. M., Dixon C. M., Barnes P. J. (1986). Effects of inhaled platelet activating factor on pulmonary function and bronchial responsiveness in man.. Lancet.

[PDF_00331] Ganbo T., Hisamatsu K. (1990). Mucosal dysfunction and damage induced by platelet activating factor (PAF).. Acta Otolaryngol.

[PDF_00351] Ganbo T., Hisamatsu K., Nakazawa T., Kamijo A., Murakami Y. (1991). Platelet activating factor (PAF) effects on ciliary activity of human paranasal sinus mucosa in vitro.. Rhinology.

[PDF_00346] Lee T. C., Malone B., Wasserman S. I., Fitzgerald V., Snyder F. (1982). Activities of enzymes that metabolize platelet-activating factor (1-Alkyl-2-acetyl-sn-glycero-3-phosphocholine) in neutrophils and eosinophils from humans and the effect of a calcium ionophore.. Biochem Biophys Res Commun.

[PDF_00319] Misawa M., Iwamura S. (1990). Platelet-activating factor (PAF)-induced rhinitis and involvement of PAF in allergic rhinitis in guinea pigs.. Jpn J Pharmacol.

[PDF_00339] Ninio E., Mencia-Huerta J. M., Heymans F., Benveniste J. (1982). Biosynthesis of platelet-activating factor. I. Evidence for an acetyl-transferase activity in murine macrophages.. Biochim Biophys Acta.

[PDF_00333] Terashita Z., Imura Y., Takatani M., Tsushima S., Nishikawa K. (1987). CV-6209, a highly potent antagonist of platelet activating factor in vitro and in vivo.. J Pharmacol Exp Ther.

[PDF_00336] Wykle R. L., Malone B., Snyder F. (1980). Enzymatic synthesis of 1-alkyl-2-acetyl-sn-glycero-3-phosphocholine, a hypotensive and platelet-aggregating lipid.. J Biol Chem.

